# Investigating the employment motivation, job satisfaction, and dissatisfaction of international high school teachers in China: the impact of the COVID-19 pandemic

**DOI:** 10.3389/fpsyg.2024.1271604

**Published:** 2024-02-07

**Authors:** Junhua Mo, Gareth Morris

**Affiliations:** ^1^School of Foreign Languages, Soochow University, Suzhou, China; ^2^School of City Culture and Communication, Suzhou City University, Suzhou, China; ^3^Centre for English Language Education, The University of Nottingham Ningbo, Ningbo, China

**Keywords:** international high school teachers, employment motivation, job satisfaction, job dissatisfaction, pandemic impact, teacher development

## Abstract

International education has become increasingly challenging to manage in an unpredictable world beset by pandemics, regional disputes, and evolving market practices. The last few decades have seen a huge demand for international education in China, and numerous acclaimed international brand names set up operations in China’s K12 schooling sector. However, the COVID-19 pandemic exerted a noticeably negative impact on international high schools and their staff in China, and exacerbated a difficult period of management for these institutions. Interestingly, but perhaps unsurprisingly, the actual operation of these educational workplaces remains under investigated in academic studies. This paper therefore attempts to examine international high schools in China by focusing on their teachers and their associated employment motivation, job satisfaction, and dissatisfaction which has been perceptibly influenced by the impact of the COVID-19 pandemic. Through a qualitative, case-study approach which adopted semi-structured interviews, an acclaimed British high school, now closed, was investigated. The study found that international high school teachers were driven by diverse work motives such as the school’s reputation, values, salary, environment, and chances for career development. Their job satisfaction was also multi-faceted, and their dissatisfaction in certain areas concerning. That is, they derived an early satisfaction from a range of facets, such as the students, class sizes, initial workloads, autonomy and collegiality. However, these early feelings were replaced by a sense of dissatisfaction and noticeable unhappiness resulting from leadership changes and subsequent management practices, increased workload, unmet employment package expectations and obligations, as well as limited professional development opportunities. This study highlights the importance of recruiting well, generating the right starting and longer-term conditions, retaining key staff and managing astutely. The work will be of interest to policy makers, investors, leaders, managers and staff alike. It will also extend educational research in the areas of teacher motivation, satisfaction and dissatisfaction, and in particular in China in international K12 settings.

## 1 Introduction

International schools are an important component of educational globalization and this area has gained increased social attention in the 21st Century where the hunt for high-caliber staff can be a continuous cycle. This has led in recent years to a huge demand for international schools worldwide with enrollment having increased 53% in the past decade (ISC Research, 2023). According to international school data for 2022 released by ISC Research (Hingston, 2022), the global enrollment rate of the K12 International School Group increased by 70% from 2017 to 2022, and this is despite a global pandemic. The growth of international schools in China has also been exponential, and in 2019 80% of schools opened by British institutions were in China (Staton, 2023). That said, this trend was affected by the COVID-19 pandemic and evolving educational policies nationwide. Thanks to the rapid development of the Chinese economy, an increasing number of Chinese families have subsequently acquired the financial ability to choose diverse educational forms and venues for their children. The large size of China’s population, its ever-growing middle class, and the resulting overwhelming examination pressure has led to an increasing number of Chinese parents considering international education as this opens up overseas university and emigration opportunities as Li (2016) highlights. It can also facilitate a smoother entry into domestic higher education. This can be seen from the trend of Chinese children starting to receive an international education from an increasingly young age. According to the 2021 Report on Chinese Students’ Overseas Study (New Oriental School, 2021), the percentage of Chinese students who plan to study in foreign middle and high schools rose from 20% in 2019 to 29% in 2021. This is an important figure given the potential student numbers involved.

With the rapid growth of international schools, teachers working for them have also become an emerging field of inquiry (Poole and Bunnell, 2023). As the mainstay of international education, these teachers are, to a large extent, expected to bring about educational success. This is especially the case with international high school teachers who are under greater pressure than their counterparts at the other stages of the K12 education to help their students to secure a high score in college entrance examinations or receive a good offer from a prestigious foreign university. Despite the important role that international high school teachers may play, they have not yet received due attention in academic investigations. Consequently, they remain largely under researched as a working demographic group. For example, what motivates teachers to seek employment in international high schools in China and to what extent they are satisfied or dissatisfied with their job are questions that are important to know for a wide range of reasons.

Thinking back to the past couple of years, and mindful of what might happen in the future, the then unexpected COVID-19 pandemic sparked a significant global educational crisis (Reyes-Guerra et al., 2021). Indeed, Staton (2023) was reporting that by the start of January 2023 the percentage of schools opened by British institutions in China had dropped to 15% highlighting just how great the impact was in this context. For the first time in decades no longer was it business as usual. For many teachers this was a period when online teaching and hybrid learning featured prominently, learner engagement proved variable and pedagogic effectiveness questionable. It was a period in time that was additionally epitomized by the emergence of a new work/life balance. As Allen et al. (2020) point out, the COVID-19 pandemic severely disrupted schooling, and placed additional demands on teachers. The pandemic also created numerous additional challenges such as how to prepare students for high stakes exams, adapt to changing domestic policies, and recruit and retain high quality staff (Nickerson and Sulkowski, 2021). For some international schools the pandemic resulted in an existential crisis, leading educational leaders to grapple with unprecedented volatility, uncertainty, complexity, and ambiguity (Kaiser, 2020). Although some studies have been carried out to investigate how the pandemic impacted international high school students in China (Shen, 2022), very few efforts have been made to study their teachers.

This study considers the case of one very high-profile British school that opened in China in 2021. Given the crucial role that teachers may play in the operation of international schools, this study is focused on the employment motivation, job satisfaction and dissatisfaction of teachers working in this British branded high school in China, and the massive impact that the COVID-19 pandemic exerted on them as it exceeded what might have been predicted. An educated guess would have expected some disruption and difficulties, but a closure and complete upheaval provided both opportunities and numerous challenges that were certainly unforeseen at the time.

## 2 Literature review

### 2.1 Motivation

Motivation refers to the driving force or stimulus that propels an individual toward a particular course of action. It has significant associations with job satisfaction and has been the subject of numerous early and contemporary theories. Some early theories include Freud’s (1949) work on the subconscious and unconscious thought processes, Hull’s (1943) drive reduction theory, and Maslow’s (1970) hierarchy of needs. These have laid the foundation for further research on individual-oriented theories, such as Vroom’s (1964) expectancy theory, Adams’ (1965) equity theory, Atkinson and Raynor’s (1974) achievement or competence theory, Bandura’s (1977) self-efficacy theory, Deci and Ryan’s (1985) self-determination theory, Locke and Latham’s (1990) goal setting theory, and Kubanyiova’s (2019) teacher self-theory. Motivation also intersects with management theory, as evidenced by Herzberg’s (1959) argument against force and his emphasis on the maintenance of incentives through his motivation hygiene theory. Additionally, McGregor’s (2006) theory posits that employees are likely to either require supervision and a more stringent management approach or will be internally motivated, which enables a softer, more participative management style.

### 2.2 Teacher motivation

**Figure 1:**
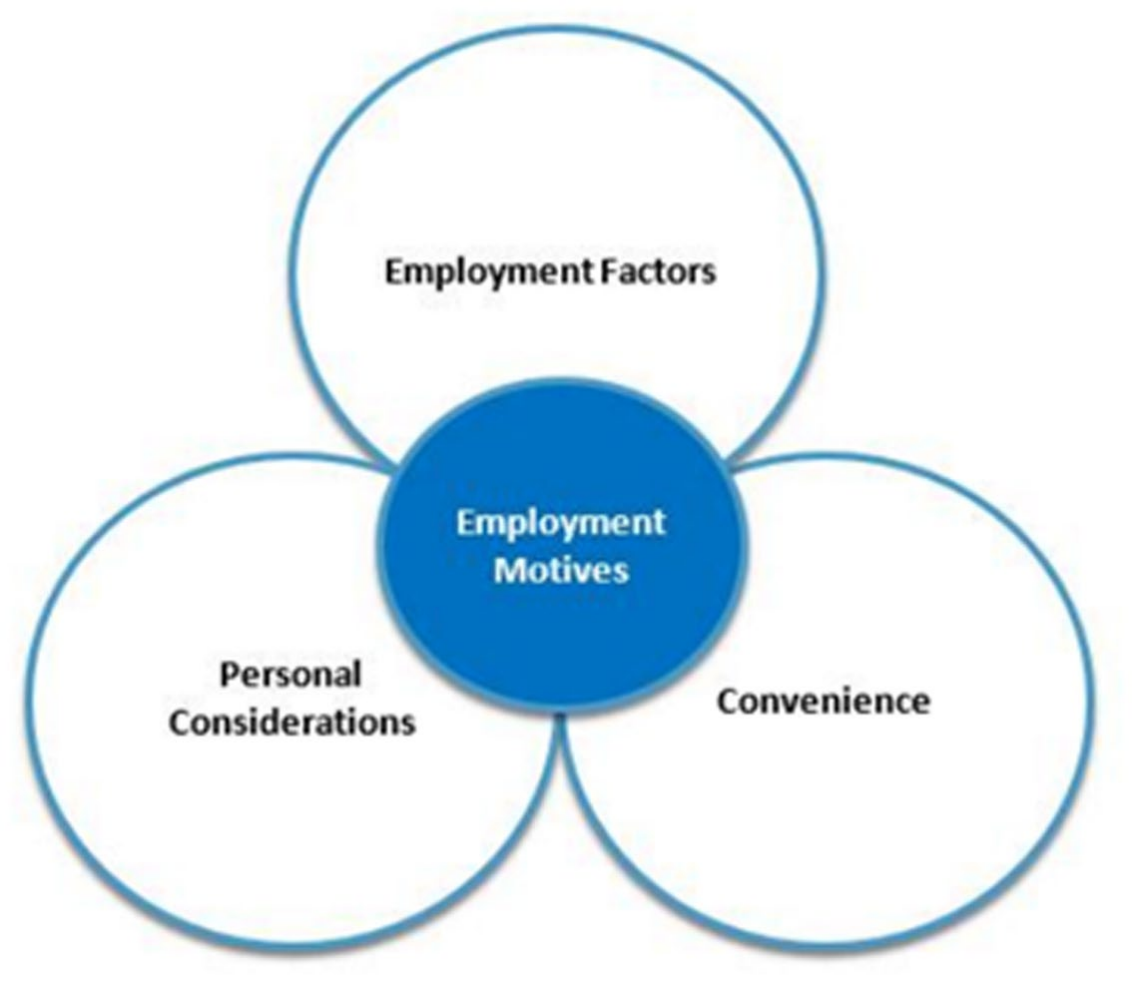
Teacher employment motivation framework (Morris, 2021).

The field of teacher motivation is understandably complex and multifaceted, as highlighted by Shoaib (2004), with influences stemming from various global, contextual, and situational factors, as well as cultural and social elements, as outlined by Vallerand and Ratelle (2002) and Ushioda (2007), respectively. Furthermore, motivations are emergent, dynamic, and multifaceted, as argued by Dörnyei et al. (2014) and Dörnyei and Ushioda (2010), with specific motives for a particular course of action easier to identify than those for prolonged activity engagement. For teachers, motives may include a range of push and pull factors, as suggested by Cai and Hall (2015), Karavas (2010), Kissau et al. (2019), and Trembath (2016). Additionally, intrinsic and extrinsic factors, altruistic motives, and non-work-related elements may impact teacher motivation, as noted by Morris (2021). For the purpose of this study, given the few theoretical constructs to comprehensively address teacher employment motivation, Morris’ (2021) framework has been adopted. He suggests that work-based motivation requires an assessment of employment-based and personal factors, as well as move convenience. Employment-based factors relate to the work, context and contract. Personal considerations consider an individual’s personal circumstances. Convenience is how easy it is to actually make the move (see Figure 1).

### 2.3 Satisfaction

In contrast to motivation, which pertains to the driving force behind our actions, satisfaction refers to our perception of past events (Carr, 2005), and the extent to which our expectations were met (Robson, 2022). It relates to a positive affective or emotional state resulting from work appraisals and experiences (Kreitner and Kinicki, 2012), with cognitive evaluations crucial in our assessment of whether actual outcomes meet or exceed our desired or expected designs, as Sharma and Jyoti (2009) suggest. Job satisfaction is a multifaceted and dynamic concept (Griva et al., 2012), as too is motivation, and its importance for teachers should not be underestimated (Judge et al., 2002), as it can impact performance (Arifin, 2015), effectiveness (Selmer and Lauring, 2011), discipline (Sungu et al., 2014), and retention (Yoshihara, 2018). Furthermore, it is linked to organizational effectiveness and retention (Morris, 2021).

### 2.4 Teacher satisfaction

Teacher satisfaction can be influenced by a plethora of factors. Altruistic and intrinsic fulfillment is one of the most important, as emphasized by Afshar and Doosti (2016). Personal interactions and relationships with co-workers and students also play a critical role in job satisfaction (Wilkins and Neri, 2019). The overall environment and community ethos are also significant contributing factors (You et al., 2015; Aldridge and Fraser, 2016). Teaching and learning aspects, such as job or task characteristics (Naumann, 1993), novelty and variety (Jonasson et al., 2017), and perceived interest (Wilkins and Neri, 2019) are highly influential as well. Additionally, autonomy, challenge, meaningfulness, responsibility, and creativity are key factors that can impact satisfaction levels (Morris, 2021). Recognition and opportunities for progression, along with the employment package, are also essential variables (Morris, 2021). Finally, non-work-related factors, such as personal relationships with loved ones, should not be overlooked, as the spillover effect can be powerful (Morris, 2021; Cacioppo, 2022).

### 2.5 Teacher dissatisfaction

**Figure 2:**
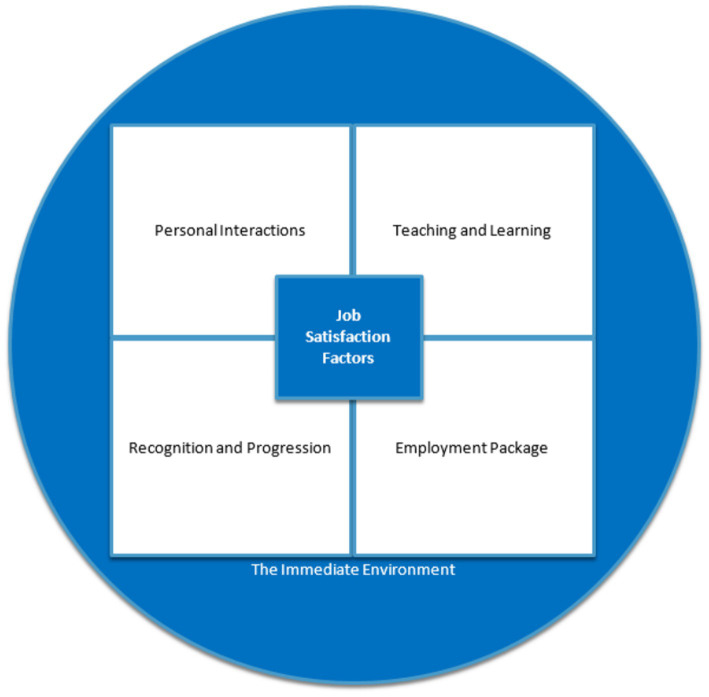
Teacher job satisfaction and dissatisfaction framework (Morris, 2021).

Similar to teacher satisfaction, teacher dissatisfaction can arise from a diverse range of factors. Morris (2021) categorizes these factors into negative personal interactions, teaching and learning factors, lack of recognition and career progression, and employment package, in addition to external personal considerations. Morris (2021) notes that disengaged students, challenging relationships with colleagues, administrators and management, and an unfavorable working environment are influential factors in personal interactions. Workload and job responsibility are typically related to teaching and learning factors, while limited opportunities for professional development, promotion, and career growth contribute to dissatisfaction concerning recognition and progression. Morris (2021) also emphasizes that working conditions, unstable job security, and perceived or actual issues are relevant. Compensation commonly features in concerns related to the employment package, while family issues and societal influences may influence external personal considerations among teachers and their families (see Figure 2).

### 2.6 Impact of the COVID-19 pandemic on teachers

Numerous studies have revealed that the COVID-19 pandemic has had a negative impact on teachers. According to Baker et al. (2021), teachers, in general, encountered more stressful than protective factors during the pandemic. Teachers found lack of connection and online teaching challenges to be the most difficult aspects of their profession. However, they identified support from co-workers and administrators as the most helpful factor in managing these challenges. Likewise, Vargas Rubilar and Oros (2021) found that the majority of teachers reported experiencing high and moderately high levels of stress during this period. The primary stress factors identified were uncertainty regarding the consequences of the pandemic, work overload, and an inadequate working environment. There was a positive correlation between perceived stress levels and the manifestation of undesired psychophysical symptoms. Furthermore, teachers with higher stress levels and more indicators of discomfort exhibited heightened susceptibility for professional burnout.

Of course, studies of pandemic impact on teachers are not all negative. There was a positive note in some studies. Sacré et al. (2023) found that despite experiencing emotional exhaustion during the pandemic, teachers expressed satisfaction with their occupation. Bailey (2021) found that although the pandemic had worsened certain aspects of precarity for international teachers, some elements of privilege persisted.

In this study, it was anticipated that the respondents would demonstrate a variety of employment motives for joining the K12 provider. The brand name, renowned reputation and a diverse and high-quality initial staffing range of profiles, alongside a competitive employment package and the opportunity to improve were likely to have been attractive pull features. It was also anticipated that despite early satisfaction given all of these elements, the fact that the school closed within a couple of years would mean that dissatisfaction would set in across a range of areas and to varying degrees.

## 3 Methodology

### 3.1 Research purpose

The purpose of this investigation was to examine the underlying motivations that drive educators to pursue careers at international high schools in China and assess the level of job satisfaction or dissatisfaction they experience in their roles. Furthermore, the study aimed to investigate the effects of the COVID-19 pandemic on these educators, who are at the vanguard of international education in China. The paradigmatic stance of this study is pragmatic, as defined by Dörnyei (2007) and Johnson and Onwuegbuzie (2004), with research design determined by the goals of the research. Ontologically and epistemologically, the study adopted a relativist position, following the ideas presented by Grix (2010), while simultaneously influenced by the principles of social constructivism expounded by Vygotsky (1978).

### 3.2 Research questions

This study sought answers to four research questions:

RQ1. What motivates teachers to seek employment in international high schools in China?

RQ2. To what extent, and in which areas, are these teachers satisfied with their job?

RQ3. To what extent, and in which areas, are these teachers dissatisfied with their job?

RQ4. To what extent, and in which areas, are these teachers impacted by the COVID-19 pandemic?

### 3.3 Research context and participants

This study was carried out in a modern affluent city in eastern China. This city is not only an economic powerhouse, but also a pioneer in international education in the country. A number of well-known international schools are also located in this metropolis.

The international high school under investigation in this study was part of a conglomerate of institutions. The institution in China had its own locally operated governance, with the UK school providing annual academic quality assurance checks, and providing advice to the investors and onsite senior leadership team.

The participants of this study were four teachers working in the international school under investigation. They were selected on a purposive premise as advocated by Creswell and Creswell (2018), with individual selection on the premise that they are willing to share their stories and express their viewpoints so as to provide insights which can help explore the concepts under evaluation at the case study institution, namely employment motivation, job satisfaction, job dissatisfaction and pandemic impact. There was also an element of convenience to the sampling as all four teachers were still accessible and had not cut off all ties to the past. The demographic backgrounds of the participants bore many similarities to other teachers at the school in that they covered a wide range of ages, experience levels and job roles and responsibilities. To maintain their confidentiality and anonymity, number codes were assigned to them when they were mentioned in this paper (see Table 1).

**Table 1:** Participant profiles.

Participant	Age	Teaching experience	Role
P1	40–50	> 15 years	Teaching and area leadership
P2	30–40	> 10 years	Teaching and area leadership
P3	30–40	> 10 years	Teaching
P4	20–30	5 years <	Teaching

P1 was a mid-career professional who had a wealth of international teaching and leadership experience. P1 was a well-qualified long-standing teacher. This was P1’s first experience of working in K12 in China. P1 was used to working in large and well-established schools.

P2 had worked in many schools in China. In one of these workplaces P2 had seen a number of affiliated international branches come and go over the course of a few years. This move was a step up.

P3 was a highly experienced teacher from the province in which the city is located. Like P2, P3 had worked in a number of schools. P3 was highly experienced with local educational practices and management approaches and coming to the school held a lot of career promise, and potentially represented a step up.

P4 was relatively new to teaching. P4 had worked in private educational businesses previously and acquired a range of valuable skills in these institutions. P4 was also a highly qualified graduate. This job was a perfect stepping stone opportunity.

### 3.4 Research methods

The present research design employed an exploratory methodology, which is deemed useful for identifying and establishing the factors and situations that influence individuals (Morris, 2021). A notable strength of this study was the opportunity for both researchers to engage with the research participants on an insider’s level, as Dörnyei (2007) highlights as advantageous. The research method selected for this study was the multiple case study approach, which is believed to offer a comprehensive, in-depth analysis and good readability (Duff, 2007). Additionally, this study employed semi-structured interviews, which are deemed beneficial in grasping interviewee perspectives and maintaining research focus (Morris, 2021). The interview guide was based on Morris’ (2021) research on the employment motivation and job satisfaction of educators in China and drew inspiration from Khan (2011). Indeed, the work of Morris (2021) contains a sample, illustrative, questionnaire in the appendix and this was designed to align to his theoretical frameworks which this study has adopted. It was also enhanced by having a follow-up data collection phase in which participant answers were further explored a few months later through discussion on points of interest. This added layers of research depth and broadened knowledge. The research also drew on authentic secondary sources of information to add further depth to the work and enhance the analytical accuracy. In this instance, ‘Glassdoor’, a well-regarded anonymous job review website, was accessed, and the insights gleaned and presented.

### 3.5 Data collection and analysis

The data of this study were collected in July 2023 by interviewing the four participants in English on an individual basis. To ensure a thorough analysis, the interviews were transcribed and a thematic analysis employed which involved data familiarization, coding, theme identification, reviewing and naming as Nowell et al. (2017) advocate. As Caulfield (2019) also suggests, a period of familiarization preceded the coding stage. The coding procedure involved a dual inductive and deductive approach, as advocated by Miles and Huberman (1994) and Sahakyan et al. (2018).

As noted earlier, a secondary data phase of data clarification and expansion in any area of interest was also undertaken which was facilitated by an additional round of exploratory questioning. The benefit of this was that it enabled the researchers to revisit and expand on areas of interest having first considered the data in its entirety and with additional insights from literature and secondary sources considered. To ensure that no concerns arose over potential inter-rater reliability, both researchers considered the data together having first analyzed the findings individually, reaching a consensus in all instances. The opportunity to check meaning and accuracy with the participants was also available throughout the research study as open lines of communication were in place.

## 4 Findings

### 4.1 Diverse employment motives

The motives for joining the international high school aligned with the employment factors, personal and convenience considerations identified by Morris (2021), but in particular it was the employment elements that were appealing. Delving further into these elements they were also multifaceted, of which the school’s reputation, values, salary, environment and chances for career development were highlighted. P2 decided to work at the school because it was a branch campus of a highly reputable UK school with a strong academic record. P2 was especially impressed with the ethos, noting:

*The school vision and values attracted me [P2].*

The values were the same as the UK institution, namely that endeavor, breadth and balance, intellectual curiosity and scholarship and respect for one another and the environment were all core beliefs. The last point emphasized the importance of a kind, caring and considerate community. P3 also was attracted by the brand name, but at the same time by the financial package.

*It was a good brand and the salary met my expectations [P3].*

In contrast, P4 saw a move as a career stepping stone. P4 actually hoped to work elsewhere but needed experience. P1 nicely summarized a more holistic evaluation which echoed the sentiments of the other staff:

*It seemed like it had an amazing potential. It had recruited high caliber teachers, had a good leadership team, and the school was affiliated to one in the UK which has a world-class reputation [P1].*

### 4.2 Distinctive job satisfaction features

Most of the teachers highlighted similar aspects of the work regarding what satisfied them. They also tended to identify the teaching and learning and personal interaction side of the job that Morris (2021) stresses as being important. P2 and P3 noted how they enjoyed seeing students improve, and knowing they played a role in this. P3 mentioned that the classes had good students and their enthusiasm and energy brightened up the days. P1 also liked teaching-related features and was content when creating and teaching good classes which promoted learning which was enjoyable. P4 added to these points, noting how preparing lessons and giving students feedback was rewarding. P4 also enjoyed seeing student progress and went on to note:

*The workload was OK. The class size was 5–1 which is good. Most students here are also good and so too is the relationship with colleagues [P4].*

Reinforcing these points, one of the two anonymous posts of the job evaluation website ‘Glassdoor’ posted in February 2023 highlighted the small size of the school (with less than 70 pupils) and classes of less than 10 as a plus. Echoing the comments about colleagues, whom P3 also felt could be nice, but building on the previous points, P1 emphasized:

*In terms of the positive aspects, most of the students were respectful, and most tried their best. The classes were also small which was great. I also had plenty of autonomy with my work, and input regarding day-to-day decision making. Good work was generally acknowledged and some of my colleagues were also very friendly and experienced. In fact, one of the main reasons I joined was because of the quality of the people on paper [P1].*

Beyond the students and staff, P3 felt that the school had a beautiful campus and time in which one could develop one’s self. P3 also felt the direct line manager provided professional development opportunities for the team. P4 also felt that the school provided opportunities in which to develop. They stated:

*I like being a teacher and I hope to be more professional and get promoted in the future* [P3].

*After finishing my master’s working here was a good choice as I could learn a lot [P4].*

For P2, the most rewarding aspects were line manager, parent and student recognition. P2 noted that in his opinion additional positive features included the working conditions and resources and effort acknowledgment, while the workload, autonomy, advancement opportunities, management support and employment package were reasonable.

### 4.3 Rise and effect of job dissatisfaction

Despite the numerous positives there were also plenty of areas in which dissatisfaction arose. Areas of dissatisfaction focused a lot on deteriorating personal interactions, alongside the employment package issues and amendments. The interviews also highlighted how dissatisfaction extended to the teaching and learning sphere and recognition and progression, another area identified by the satisfaction and dissatisfaction framework of Morris (2021). P2 noted that dissatisfaction grew quickly despite early job satisfaction.

*When I first joined the school I was quite satisfied with my job and career, but due to the frequent changes in senior management and the working environment I’m not satisfied with my job now [P2].*

P3 had thoughts on the employment package:

*I think the salary is OK, but I cannot afford a decent life [P3].*

P1 stressed the role that negative interactions can have, noting a dislike for situations in which the workplace was not friendly, people acted aggressively, or when people were simply being difficult. P1 added that uninterested students were also dispiriting before concluding:

*On the flip side, some of my colleagues became very unhappy early on with what the working reality morphed into, especially after two senior figures left during the first semester. This led to numerous staff suffering from anxiety, unhappiness, and stress [P1].*

P2 continued the theme about personal interactions highlighting that some of the students created more trouble than was normal. P2 was also unhappy with his treatment at the hands of one of the leadership figures:

*Sometimes I feel under privileged because one of the senior managers is very aggressive and critical [P2].*

Both the February and June 2023 ‘Glassdoor’ reviews stressed equal concerns. The first highlighted that management at the school had been inexperienced and arrogant, and that there were no clear lines of responsibility at the site. It also suggested that there were no standardized policies and procedures enacted in reality. Coupled with sudden and changing workloads, no professional development opportunities, and poor inter-school relationships born out of fear of upsetting the investors, a budget absence, shrinking staff and student numbers, the suggestion was to close the school. P4 and P1 also drew attention to some of these points:

*The leadership gives me too many lessons a day to teach which makes me tired. I would have additionally liked to have refused work arranged for the weekend, but it might not have been appropriate. I’m also not satisfied that I had no medical provision, and I would like to have had more professional development opportunities. Ultimately, the job here is not stable and I do not have promotion opportunities [P4].*

*The workload became excessive. It just kept building with staffing cuts, and this led to burnout in some cases. There was also very little funding and resources were scarce and only got scarcer despite the early suggestions that these were not issues. This had an impact on everything from professional development opportunities which disappeared as the funding wasn’t there, to job security. As cuts came, people were not always replaced, and jobs moved from full time to part time. It felt like a revolving door at times. It was sad because by the end trust was completely eroded and the environment oscillated from toxic and fractious to despondent and apathetic [P1].*

The second ‘Glassdoor’ review was even more damning. It claimed horrendous mismanagement was taking place in July 2023, and that the school was closing. It claimed that the sister school had been aware of concerns in the area of health and safety, safeguarding and staff mistreatment with lawsuits against former students and also those filed by former employees known for trying to leave a failing school or for failing to adhere to legal obligations. It finished with an even stronger rebuke which likely led to this post’s later removal.

P2 echoed some of these concerns mentioning the evaluation and promotion of staff, the job security and the staff and management collegiality was not good. The omission of a medical examination was also irresponsible. More concerning was when P2 stated:

*I would not recommend this school to anyone else because it’s no longer like it used to be after so many changes [P2].*

P3 added that as a private institution the work was unstable, and job security was problematic:

*I do not feel secure in my work environment because of some bad management. In the beginning it was a good brand and we had professional leaders so the teachers were motivated and active. The principal was nice and supportive during the journey. Now the school is going to shut down and I feel insecure [P3].*

P3 added that they would not recommend the school, and that there had been times when they did not want to be there anymore.

### 4.4 Severe impact of the COVID-19 pandemic

All of the staff participants stated that they felt that the pandemic had influenced subsequent developments. P2 stressed the impact that it had on his teaching and the need for training in order to adapt and upskill:

*I needed to spend more time searching for teaching materials, mastering course skills and revising lesson plans. I also needed to adjust my teaching methods and learn how to organize online teaching and use different software and apps [P2].*

P3 also highlighted the importance of training and time in developing the new skills to be mastered during the pandemic. P3 mentioned how these had built up over time enhancing competencies and resilience, but felt it had taken an emotional and psychological toll:

*At first the pandemic caused me anxiety because I could no longer go to school and follow my usual work schedule on campus. The virus also made us feel unsafe and insecure both mentally and physically. If there had been lectures guiding us about how to maintain mental health it would have been much better. The online lessons helped ease the anxiety in me because the technology made it possible to connect with students and colleagues. However, the need to use the new technology and fulfil my teaching requirements also made me anxious. If there had been more training I believe it would have made life easier [P3].*

P1 summarized the impact more broadly on a holistic operational level:

*The pandemic almost certainly had a massive financial impact which meant that the school was always playing catch up. The cost cutting that ensued in an attempt to better balance the books almost certainly also led to the rapid decline of the institution because quality inevitably suffered, trust went, tempers frayed and people voted with their feet. Teaching online had some advantages at times as it saved on a long daily commute. That being said, learning suffered. I feel that this job is really a case of what might have been because the school is closing, and it feels like a massive missed opportunity given how successful the school might have been had things been done differently and different decisions made at certain junctures. This could have been an amazing school. What a missed opportunity [P1].*

## 5 Discussion

### 5.1 Pull factors as a powerful motivational force

A number of factors proved influential in motivating these staff to seek employment at this particular institution initially. The first was the power of the UK sister school’s brand and values. This struck a chord with the majority of the participants in this study, as P1, P2, and P3 mentioned. A brand promise that identifies who a group of people are and what they stand for collectively is regarded as an expression of a company’s vision and acts as a powerful pull motive due to the emotional engagement it can generate as Barlow (2019) highlights. Many people want to work for well-regarded employers and this one carried a powerful name within education circles. Part of the explicit appeal also lay in the values which promoted respect and kindness. The importance of kindness to individuals is well documented as Ferrucci (2007) detailed, and working within a respectful environment is also important to educators both generally and in the city in which this study took place, as the work of Morris (2021) highlighted. There was also the potential implicit, and subconscious, motivational allure of working for an employer that could enhance individual “mianzi” or face, as well as act as a useful career stepping stone. Beyond the promise of what the employer potentially stood for, and the perceived alignment between personal and professional values, additional employment factors were also deemed to be important. The financial package was competitive, albeit nothing too exciting as far as P3 was concerned, and opportunities for career advancement were in evidence as P4 alluded to. These are important points among a multitude as Morris and Mo (2023) stressed. Finally, the opportunity to work with what appeared to be an exciting team of professionals was another motivational pull factor.

### 5.2 Early job satisfaction existing in numerous areas

There is a wide array of factors which can satisfy employees once they begin to work for an employer, and in this case study numerous overlaps with existing findings were evidenced. Zembylas and Papanastasiou (2006) highlighted the importance that making a social contribution can have, and all of the teachers derived a sense of happiness in helping the students they taught to progress. Wilkins and Neri (2019) also explicitly drew attention to the importance that personal interactions and associated relationships can have for job satisfaction, and the value placed on collegiality between colleagues was also mentioned by all of the staff. P4 and P1 stated how pleasant it was initially to work with friendly teachers and administrative staff, and P3 mentioned how he got on well with his line managers. Beyond personal interactions, a number of teaching and learning features were also well regarded. The small class sizes were a source of contentment, although how sustainable they were from a financial standpoint was debatable, as bespoke sessions of one or two were an excellent advertising feature, but also potentially suggested a recruitment concern despite the relatively high student fees. Being able to be creative was also important, and Morris (2021) collated a range of teaching and learning elements which satisfy staff, among which is autonomy, agency and trust, alongside an ability to have one’s voice heard as far as decision making is concerned. The traditional, small but green campus also appealed. Recognition and progression in contrast was less of a source of work satisfaction, although for early career professionals like P4 opportunities were available sooner than staff who might have experienced them elsewhere had the school been more stable. The importance of career opportunities is mentioned by Jonasson et al. (2017). Having some time for professional development was another attractive feature, but this was more because of the work diversity and variety rather than the number of free hours in the working day. Despite the salary not being overly praiseworthy as far as P3 was concerned, for expatriate staff, as P1 alluded to, the employment package was competitive on paper.

### 5.3 Changes to be staged and managed carefully

Despite the fact that the staff were satisfied by a variety of employment features covering a range of areas, it was also evident that this initial satisfaction declined over time for some, as dissatisfaction grew in others. This might have been due to expectations, some potentially subconscious, no longer being met, but it had a powerful and detrimental impact as Robson (2022) draws attention to. Perhaps the area of the biggest concern was in terms of personal interactions, and most notably senior management as P2, P3 and P1 noted. Almost certainly there was a sense of evolving situation and context not aligning with the anticipated reality, which, as Robson (2022) noted, shape our experiences. Some senior management figures were used to working in European international schools with experienced practitioners where the expectations were very clear. Other leadership figures came from a more localized management mindset in which total subordination was expected, which upset practitioners used to being, and wanting to be, treated with respect. The motivational lessons of inspirational leadership advocated by Sinek (2017), and indeed Carnegie (2022), went unheard as time progressed and pressure mounted and default positions were reverted to. The aggressive and critical leadership that P2 noted partially contributed to the feelings of unhappiness and anxiety P1 mentioned. Teaching and learning changes also led to dissatisfaction. The seemingly ever-increasing workload as P4 and P1 discussed, and anonymous job site reviews, the employment package not being honored, and the absence of a medical insurance for locally recruited staff all added to the sense of discontent and frustration. The loss of professional development opportunities and funding, and a lack of promotion pathways meant that progression and recognition also faltered. This ran counter to the expectations of staff, which are a highly powerful emotive feature as Robson (2022) draws attention to. With little to endear the school publicly, and with an internal sense of fractiousness, toxicity and apathy, as P1 alluded to, the difficult task of navigating a period of policy changes and pandemic challenges fast became impossible.

### 5.4 The pandemic legacy requires smart navigation

Undoubtedly, the pandemic had a significant impact on this case study institution as it delayed the opening by an academic year, meaning a period of lost revenue resulted. Consequently, a second round of staffing recruitment was necessitated and left a number of practitioners unhappy about early cost cutting which led to numerous administrative employees being made redundant. Many accounts of the pandemic discuss the impacts on students’ lost education and the legacy of this, as Broom (2022) illustrates. Other works consider the far-reaching impact on the education industry of this period, including increased educator workloads, mental health and stress level increases, digital skills gap, and funding deficits (Staples., 2021). For private investors the pandemic tested the ability and desire to either play the long game or short one, with rapid cost cutting a risk with a brand built around quality and stability. The lack of senior leadership retention in this situation as lockdowns wore on, increasingly prevalent cuts, and an inexperienced and heavy-handed management style caused, in part, by the situation also exacerbated the issues. So too did the difficulties presented by the sister school not having any real oversight over proceedings, as the study participants alluded to. Inevitably, policy changes were also influential on the eventual school closure, but like many developments were not insurmountable obstacles in themselves as other business enterprises proved.

## 6 Conclusion

### 6.1 Major findings and implications

This study has considered the employment motives, job satisfaction and dissatisfaction of four international high school teachers at a British branded institution in China. The study has highlighted that a variety of motivational forces influenced these educators. Some notable motives included the brand, its purported values, the employment package, the people and the potential. When employed at the school early satisfaction was derived from a range of facets, such as the students, class sizes, initial workloads, autonomy and collegiality. However, the results also show these early feelings were replaced by a sense of dissatisfaction over time and noticeable unhappiness by the end. Dissatisfaction resulted from leadership changes and subsequent management practices, increased workload, unmet employment package expectations and obligations, as well as limited professional development opportunities and resources became increasingly scarce. The outbreak of the COVID-19 pandemic also had a detrimental effect on international high school teachers in China as the educational business landscape was adversely affected, increasing their negative experiences, a point to which these teachers alluded.

This research suggests a number of recommendations based on the findings and through consideration of the literature. First, the diverse employment motives of international high school teachers should be recognized. And there is also often an extrinsic consideration as Munyengabe et al. (2017) draw attention to. Second, the finding that international high teachers’ job satisfaction ebbed away while job dissatisfaction mounted with the passing of time should serve as a cautionary tale for businesses, educational providers, leaders and educators alike. Thirdly, the severe impact of the pandemic on international high school teachers should be given due acknowledgement by policy makers, investors, school administrators and staff alike.

### 6.2 Limitations and future research directions

Inevitably, there were some limitations in this study. Firstly, the size of the data was not sufficient to provide a panoramic view of work-related issues faced by international high school teachers. This study only investigated a single institution and a small number of teachers. This is, of course, attributive to the exploratory nature of the case study, which was well justified. Secondly, the research method mainly comprised of qualitative interviews, despite the fact that this study also considered comments left on external job sites such as ‘Glassdoor’. Consequently, the methodological approach of this study was largely unitary. In addition, the research considered the motivation, satisfaction and dissatisfaction based on the explicitly stated responses of the teachers. It is possible that there were implicit, likely subconscious, drives and factors that also influenced the participants, and their feelings and perceptions, but to explore this would expand the scope of this work beyond what is possible in this study.

There are a number of possibilities for future research. Firstly, it would be good to broaden the scope and collect more information from additional K12 providers and their academic teaching teams. Studies could also investigate administrative staff’s perspectives. Secondly, future research in the same area, and with similar participants, could also utilize different or additional data collection approaches based on this exploratory study’s insights. For example, a quantitative approach such as a large-scale survey will generate a more holistic view. It could also delve into complementary fields, such as leadership and psychology, and explore alternative perspectives and possible contributory factors. Thirdly, research could also be extended to other international high schools in China, not necessarily linked to a British brand or located solely within the immediate locality, with more voices being heard. Additional temporal moments could also be considered. In brief, it would be good to broaden the scope and expand the method so as to gain more insights into the work and minds of teachers working in the international high schools in China and, indeed, throughout the world.

## Data availability statement

The original contributions presented in the study are included in the article/Supplementary material, further inquiries can be directed to the corresponding author.

## Ethics statement

The studies involving humans were approved by the Department Ethics Committee of Soochow University, Suzhou, Jiangsu. The studies were conducted in accordance with the local legislation and institutional requirements. The participants provided their written informed consent to participate in this study. Written informed consent was obtained from the individual(s) for the publication of any potentially identifiable images or data included in this article.

## Author contributions

JM: Writing – original draft. GM: Writing – original draft.
