# Supplementary material for: Investigating the employment motivation, job satisfaction, and dissatisfaction of international high school teachers in China: the impact of the COVID-19 pandemic
Source: Front Psychol. 2024 Feb 7;15:1271604. doi: 10.3389/fpsyg.2024.1271604 (PMC10879565; doi:10.3389/fpsyg.2024.1271604)
Supplement: Supplementary file 1 [file Table_1.DOCX]

**Investigating the employment motivation, job satisfaction and dissatisfaction of international high school teachers in China: The impact of the COVID-19 pandemic**

**Interview guide**

1. How/why did you decide to work at this school specifically?

2. What are some of the things that could make your day in your current job?

3. In general, how do you feel about your job and career?

4. Are you satisfied with your job and career? Why/Why not?

5. What are some of the things that could ruin your day in your current job?

6a. How do you feel about working in your school?

6b. Has the COVID pandemic had any impact on how you feel?

7. What aspects of teaching or work related activities do you find most rewarding?

8. What aspects of teaching or work related activities do you find frustrating?

9a. How has your experience been overall working at this institution/school?

9b. Has the COVID pandemic had any impact on how you feel?

10. Would you recommend this institution to anyone else?

11. Have you ever been in a situation where you decided that you didn’t want to continue working here anymore?

12. How long do you plan to work here?
